# Jugular Venous Reflux Is Associated with Perihematomal Edema after Intracerebral Hemorrhage

**DOI:** 10.1155/2017/7514639

**Published:** 2017-06-11

**Authors:** Hao Feng, Hongxia Zhang, Wen He, Jian Zhou, Xingquan Zhao

**Affiliations:** ^1^Department of Neurology, Beijing Tiantan Hospital, Capital Medical University, Beijing 100050, China; ^2^China National Clinical Research Center for Neurological Diseases, Beijing 100050, China; ^3^Center of Stroke, Beijing Institute for Brain Disorders, Beijing 100050, China; ^4^Beijing Key Laboratory of Translational Medicine for Cerebrovascular Disease, Beijing 100050, China; ^5^Department of Diagnostic Ultrasound, Beijing Tiantan Hospital, Capital Medical University, Beijing 100050, China; ^6^Department of Radiology, Beijing Tiantan Hospital, Capital Medical University, Beijing 100050, China

## Abstract

The purpose of this study was to determine whether jugular venous reflux (JVR) is associated with perihematomal edema (PHE) in individuals with intracerebral hemorrhage (ICH). Patients with spontaneous supratentorial ICH within 72 h of symptom onset were enrolled. Baseline brain computed tomography (CT) scan was performed, with a follow-up CT examination at 12 ± 3 days after onset. Jugular venous color Doppler ultrasound was performed at 12 ± 3 days after onset to examine the JVR status. A total of 65 patients with ICH were enrolled. In logistic regression analysis, absolute PHE volume was significantly associated with JVR (OR, 5.46; 95% CI, 1.04–28.63; *p* = 0.044) and baseline hematoma volume (OR, 1.14; 95% CI, 1.03–1.26; *p* = 0.009) within 72 h of onset. It was also correlated with JVR (OR, 15.32; 95% CI, 2.52–92.99; *p* = 0.003) and baseline hematoma volume (OR, 1.14; 95% CI, 1.04–1.24; *p* = 0.006) at 12 ± 3 days after onset. In a similar manner, relative PHE volume was significantly associated with JVR (OR, 14.85; 95% CI, 3.28–67.17; *p* < 0.001) within 72 h of onset and at 12 ± 3 days after onset (OR, 5.87; 95% CI, 1.94–17.77; *p* = 0.002). JVR is associated with both absolute and relative PHE volumes after ICH.

## 1. Introduction

Intracerebral hemorrhage (ICH) is a neurological disease with mortality rates within 30 days reaching 30–52% [1–3]. Besides the initial hematoma volume, subsequent growth and other factors such as age, intraventricular blood, and perihematomal edema (PHE) also contribute to poor outcome [4, 5]. Indeed, PHE is considered an important prognostic factor in patients with ICH [6]. As part of secondary brain damage in ICH, PHE begins to develop within the first 24 h of onset [7] and grows rapidly in the first three days, peaking about two weeks later [8].

Jugular venous reflux (JVR) is widely found in healthy individuals, with a prevalence of about 20–40%, which increases with age [9]. JVR is characterized by a retrograde flow in the internal jugular veins (IJVs) during Valsalva-like maneuvers (VM) or spontaneously [10]. Previous studies have demonstrated that JVR can retrogradely transmit hypertension into the cerebral venous system and is associated with structural changes in the brain parenchyma of patients with mild cognitive impairment and Alzheimer's disease, increasing vasogenic edema in the brain [10, 11]. Preliminary data showed that cranial venous outflow abnormalities increase brain edema after arterial ischemic stroke [12]. Based on the above findings, we hypothesized that JVR increases PHE volume after ICH.

To test this hypothesis, this cohort study was carried out with the aim to assess the association of JVR and PHE volume.

## 2. Methods

### 2.1. Patients

This was a cohort study assessing patients with spontaneous supratentorial intracerebral hemorrhage admitted to the Department of Neurology, Beijing Tiantan Hospital, China. The inclusion criteria were (1) age ≥ 18; (2) admission diagnosis of supratentorial ICH confirmed by brain computed tomography (CT); and (3) baseline CT scan completion within 72 h of onset. The exclusion criteria were (1) infratentorial hemorrhage; (2) primary intraventricular hemorrhage; (3) subsequent surgery; or (4) any suspected cause of secondary ICH. Data were prospectively collected and retrospectively analyzed. This study was approved by the Ethics Committee of Beijing Tiantan Hospital affiliated to the Capital Medical University of China, in compliance with the Declaration of Helsinki. All patients or their legal representatives provided signed informed consent.

### 2.2. Collection of Patient Baseline Characteristics

Eligible patients were assessed for demographics data, clinical data, and hematoma and PHE volumes. Blood samples were collected from each patient on the day of hospital admission for the measurement of blood glucose (GLU), low-density lipoprotein (LDL) levels, and international normalized ratio (INR). Clinical and neurological evaluations were performed to collect data, including first blood pressure measurement after onset, as well as history of smoking, hemorrhagic and ischemic stroke, diabetes mellitus, hypertension, coronary heart disease, and blood diseases (including coagulopathy and leukemia). History of smoking was defined as currently smoking or past smoking (i.e., no smoking for the past 5 years). Arterial hypertension was defined as systolic blood pressure ≥140 mmHg and/or diastolic blood pressure ≥90 mmHg, or self-reported current treatment for arterial hypertension with antihypertensive medications. On the admission day, the patients were assessed for stroke severity, consciousness state, and the level of handicap according to the National Institute of Health Stroke Scale (NIHSS), Glasgow Coma Scale (GCS), and modified Rankin Scale (mRS), respectively.

### 2.3. Color Doppler Ultrasound

**Figure 1:**
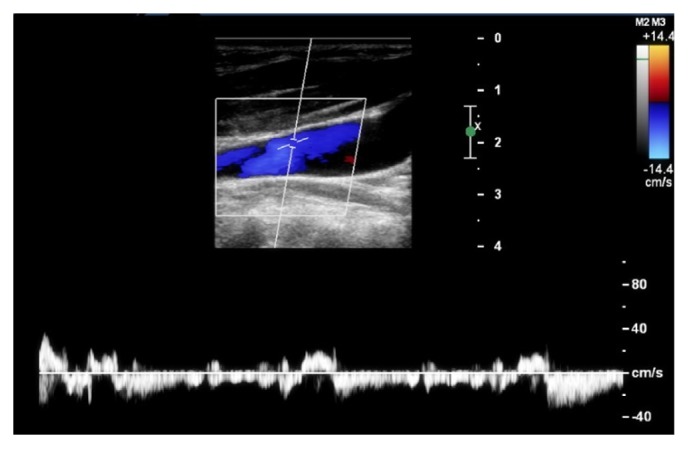
Retrograde flow detected in the Doppler spectrum spontaneously is considered jugular venous reflux (JVR).

Color Doppler ultrasound was carried out on GE Logiq9 (General Electric, Fairfield, USA), with the patient lying flat in the supine position and the head straight, using a 4–7 MHz linear transducer after a 10 min of quiet rest. All examinations were performed by an experienced neurosonographer blinded to the patient's clinical data. Sufficient ultrasound gel was applied and appropriate care was given to avoid compression on the neck veins during examination. Bilateral internal jugular veins (IJVs) were examined initially through longitudinal and cross-sectional views from the proximal part of the neck base rostrally to the distal part at the submandibular level to detect spontaneous JVR. The color box was adjusted to include the entire IJV lumen. When retrograde color appeared in the lumen center, the retrograde flow was confirmed by Doppler spectrum. JVR was defined as the retrograde flow >0 seconds detected at the distal part of the IJV (above the inferior jugular bulb) (Figure 1) [11, 13, 14]. Patients with JVR on neither side were classified as JVR-negative, while those with JVR detected on either or both sides simultaneously were considered to be JVR-positive. We also detected structural abnormalities of the IJVs as potential causes of JVR [15, 16]. All structural abnormalities were classified into two subcategories: intraluminal structural (web, flap, septa, membrane, and malformed valve) and extraluminal structural (stenosis and annulus) [15].

### 2.4. Computed Tomography

Baseline noncontrast computed tomography (NCCT) was performed within 72 h of onset and repeated at 12 ± 3 days, on a CT scanner (General Electric), with 512 × 512 matrix, FOV of 15 cm, and slice thickness of 9 or 10 mm (supratentorial) and 4.5 or 5 mm (infratentorial). A roundish or ellipsoid hematoma with smooth margin was classified as regular: one with a pleomorphic contour and several adjacent but separated hematomas, and multicentric hematomas were classified as irregular. Intracranial hematoma (excluding ventricular hemorrhage) and PHE volumes were independently measured by two trained neurologists blinded to clinical data. Images were processed off-line with the Picture Archiving and Communications Systems (PACS). The examiner manually drew regions of interest (ROIs) by tracing the hyperdense area (hematoma) and hypodense region surrounding the hematoma (PHE) throughout the lesion [17]. Threshold ranges for hematomas and edemas were 44–100 and 5–33 Hounsfield units, respectively. Hematoma volume for each slice was then calculated by multiplying the hyperdense area by slice thickness. Hematoma volumes from all slices were added to obtain total hematoma volumes. Similarly, total lesion volumes (hyperdense + hypodense) were calculated. Absolute PHE volume was measured by subtracting the hematoma volume from total lesion volume. Relative PHE was calculated by dividing the absolute PHE volume by that of hematoma. When PHE was too small to measure, a value of zero was assigned for both absolute and relative PHE volumes [17]. The subjects were grouped based on average absolute and relative PHE volumes, into the large-volume (greater than average PHE volume) and small-volume (less than average PHE volume) groups, respectively.

### 2.5. Statistical Analysis

SPSS for Windows, version 16.0 (IBM, Armonk, NY, USA), was used for all statistical analyses. Demographic and clinical data of patients (with or without JVR) were assessed by Student's* t*-test or Wilcoxon rank-sum test for continuous variables. The chi-square test or Fisher's exact test was used to compare categorical variables. Multivariate analysis was performed using a logistic regression model to screen risk factors for large PHE. Variables with* p *< 0.20 were entered in the multivariate analysis, and odds ratios (ORs) and 95% confidence intervals (CIs) were calculated. Two-sided* p *< 0.05 was considered statistically significant.

## 3. Results

### 3.1. Patient Baseline Characteristics

A total of 65 patients were enrolled in this study. Among them, 11 (16.9%) and 7 (10.8%) subjects had right- and left-sided JVR, respectively; 11 patients (16.9%) had bilateral JVR, while 36 subjects (55.4%) showed no JVR. Among 29 JVR-positive patients, there were 10 subjects who had retrograde flow in their IJVs with a duration <0.5 s (0.11–0.49 s). The patients with right-sided, left-sided, and bilateral JVR were assigned to the JVR-positive group; those with no JVR were classified into the JVR-negative group.

No extraluminal structural abnormality was found in any subject. In the JVR-negative group, one patient had a dysfunctional valve in the left IJV and one patient had single leaflet valve in the right IJV. Among patients with left-sided JVR, two had single leaflet valve in the left IJV and one had single leaflet valve in the right IJV. Among patients with bilateral JVR, one had single leaflet valve in the left IJV and one had dysfunctional valve in the right IJV. No web, flap, septa, or membrane was observed. The baseline characteristics of the patients are shown in Table 1. No statistically significant differences were found in demographic and baseline clinical parameters between the two groups.

### 3.2. JVR Increases PHE Volumes in ICH Patients

To characterize the relationship between JVR and PHE in ICH patients, PHE volumes were compared between the two groups within 72 h of onset and at 12 ± 3 days after onset. Interestingly, both absolute and relative PHE volumes in the JVR-positive group were significantly higher than those of the JVR-negative group (Table 2).

### 3.3. Association of PHE and JVR

To further explore the relationship between PHE and JVR, the subjects were further grouped based on average absolute and relative PHE volumes, into the large-volume (exceeding average PHE volume) and small-volume (less than average PHE volume) groups (Table 3). Interestingly, the JVR prevalence in the large-volume group was significantly higher than that in the small-volume group.

### 3.4. Association of PHE Volume with Clinicopathologic Parameters 

Logistic regression analysis was carried out for each of the four conditions shown in Table 3. In univariate analyses, to avoid missing relevant variables, an association was considered statistically significant at* p* < 0.20. Absolute PHE volume within 72 h of onset was significantly associated with JVR (*p* < 0.05), sex (*p* = 0.173), smoking history (*p* = 0.135), hematoma shape (*p* = 0.095), NIHSS on admission (*p* < 0.001), mRS on admission (*p* < 0.05), GCS on admission (*p* < 0.05), baseline hematoma volume (*p* < 0.001), first systolic blood pressure (*p* = 0.189), and first diastolic blood pressure (*p* < 0.05). Meanwhile, relative PHE volume within 72 h of onset was significantly associated with JVR (*p* < 0.001), hematoma shape (*p* < 0.05), NIHSS on admission (*p* = 0.171), GLU (*p* = 0.128), baseline hematoma volume (*p* < 0.001), first systolic blood pressure (*p* = 0.189), and first diastolic blood pressure (*p* < 0.05). Furthermore, absolute PHE volume at 12 ± 3 days after onset was significantly associated with JVR (*p* = 0.001), sex (*p* = 0.005), hematoma shape (*p* < 0.05), NIHSS on admission (*p* = 0.008), mRS on admission (*p* = 0.071), GCS on admission (*p* = 0.041), baseline hematoma volume (*p* < 0.001), and LDL (*p* = 0.051). Moreover, relative PHE at 12 ± 3 days after onset was significantly associated with JVR (*p* = 0.001).

In multivariate logistic regression analysis, PHE volume remained significantly associated with JVR in all four conditions. JVR and baseline hematoma volume showed significant associations with absolute and relative PHE volumes, within 3 and 12 ± 3 days of onset, respectively, as shown in Table 4.

### 3.5. Analysis of the Threshold for JVR

The patients were regrouped according to the duration of JVR. Patients with JVR below 0.5 s were considered JVR-negative because JVR below 0.5 s was considered normal in some previous studies [10, 14, 18–24]. In the multivariate analysis, the OR of JVR was 7.35 (95% CI: 1.15–47.20;* p* = 0.035) for 72 h absolute PHE; OR = 7.15 (95% CI: 1.64–31.15;* p* = 0.009) for 72 h relative PHE; OR = 17.46 (95% CI: 3.21–94.96;* p* = 0.001) for absolute PHE at 12 ± 3 days; and OR = 6.89 (95% CI: 2.12–22.46;* p* = 0.001) for relative PHE at 12 ± 3 days. These results are similar to those obtained when using JVR > 0 s as the threshold for JVR.

## 4. Discussion

This study demonstrated that PHE volumes were significantly higher in ICH patients with JVR compared with the JVR-negative group. In addition, JVR was associated with both absolute and relative PHE volumes in these patients after adjusting for age, sex, first blood pressure measurement after symptom detection, baseline hematoma volume, hematoma shape, GLU, LDL, NIHSS, GCS, and mRS on admission.

It is admitted that hematoma volume is the main factor that determines the volume of PHE [8, 25]. In this study, baseline hematoma volumes in the JVR- positive group were similar to those of JVR-negative patients. Additionally, differences in other clinical factors that may affect PHE volume (such as age, gender, GLU, NIHSS, GCS, and history of smoking, hypertension, and stroke) were not statistically significant between the two groups. These findings suggested that JVR status, as the only difference between the two patient groups, may play an important role in the formation of PHE. This is the first study demonstrating that JVR is associated with PHE after ICH.

Venous flow from the superficial and deep venous system drains into the transverse sinus, then into the sigmoid sinus, and finally into IJV; indeed, IJVs collect most of the cerebral venous blood, especially in the supine position [19, 26, 27]. Therefore, it is quite plausible that hemodynamic changes in IJVs might alter cerebral venous drainage.

Previous studies have shown that JVR in the jugular venous system can cause retrograde hypertension, which can be transmitted to the cerebral venous system [10, 24, 28, 29]. As shown above, JVR was detected in patients at rest, indicating the retrograde venous pressure may be sustained or repetitive. Such sustained or long-term repetitive retrogradely transmitted venous pressure may subsequently lead to cerebral venous hypertension. As a result, cerebral venules and capillaries dilate [18] and blood brain barrier (BBB) permeability increases, causing large plasma molecules to leak into the brain tissue [10]. One such molecule is endothelin-1 (ET-1), a potent vasoconstrictor peptide derived from vascular endothelial cells, which was shown to synergize with JVR in causing cough syncope/presyncope [14]. The molecules leaking through the BBB subsequently increase osmotic pressure in the brain tissue [10]. These changes cause water molecules to move from blood vessels to the brain tissue, thereby promoting vasogenic edema formation [30]. Retrograde hypertension in the cerebral venous system, on the other hand, can obstruct cerebrospinal fluid (CSF) circulation and increase intracranial pressure (ICP) [14]. In patients with ICH, the hematoma itself can lead to elevated ICP, which may be aggravated by JRV; the enhanced ICP subsequently reduces cerebral blood flow (CBF) and cerebral perfusion pressure (CPP) [14]. Evidence has shown a decreased perfusion area around the hematoma after ICH; meanwhile, continuous CBF and CPP reduction results in an ischemic area around the hematoma, in which BBB is destroyed alongside increased vasogenic edema [31, 32].

In this study, JVR was detected before ICH. It remains unclear why patients with JVR seemed to be normal before ICH. The intracranial venous system contains about 70% of blood in the cerebral circulation, and ICP can be regulated effectively by the fluctuation of venous blood volume under normal conditions [20, 33–39]. With an elevated ICP, however, ICP regulation becomes ineffective so that the abnormal venous outflow contributes to increased venous congestion in patients with high ICP [20, 39]. A study of ischemic stroke reported in 2009 also confirmed that previous intracranial venous outflow hypoplasia or occlusion may induce early fatal edema after large middle cerebral artery infarction [12].

Most previous studies defined JVR as JVR > 0.5 s [10, 14, 18–24], while the present study used any JVR (or JVR > 0 s) as the definition for JVR. The exact threshold value for JVR still remains to be clearly defined, but the present study showed that using either >0 s or >0.5 s resulted in similar results in the multivariate analysis.

JVR is a form of functional abnormality of IJV and is usually observed in conjunction with chronic cerebrospinal venous insufficiency (CCSVI) and internal jugular valve insufficiency (IJVI) [21–23, 40, 41]. The origin of this phenomenon could be the congenital impairment of an IJV valve such as the absence of an IJV valve and acquired dysfunction of an IJV valve, which may be associated with tricuspid valve regurgitation and primary pulmonary hypertension [42, 43]. Tricuspid valve regurgitation and primary pulmonary hypertension could cause elevated central venous pressure leading to damaged IJV valve. Dolic et al. [16] studied 240 healthy individuals and found that the presence of heart disease (especially heart murmurs), obesity, and cigarette smoking were associated with an increased prevalence of intraluminal structural abnormalities such as malformed valve. They inferred that JVR was a secondary effect of intraluminal structural abnormalities [16]. In the present study, there was only a trend of higher percentage of malformed valve in JVR-positive subjects (17.2%) compared with JVR-negative subjects (5.6%) (*p* = 0.268). Other possible cause of JVR should be further explored in future studies.

There are two forms of JVR:VM-induced and spontaneous types [44]. In previous studies, VM was performed by forcible expiration and patients were asked to maintain a Valsalva pressure of 40 mmHg for at least 10 s [10, 14]. However, in the present study, most patients with ICH were not able to perform VM and we only detected spontaneous JVR.

Inevitably, this study had some limitations. (1) We focused solely on JVR, a hemodynamic change in the internal jugular vein, without considering other intracranial venous abnormalities. It is unknown whether such venous abnormalities participated in the formation of perihematomal edema in this study. Thus, future research involving a comprehensive evaluation of intracranial and extracranial venous system is strongly needed. (2) The relatively small sample size of this study should be mentioned. (3) For patient safety, only subjects from the general wards in our institution were enrolled, whose baseline hematoma volumes are usually relatively small. Patients in neural intensive care unit with larger hematoma volumes should be also enrolled in future studies under closer monitoring.

## 5. Conclusions

The current findings demonstrated that JVR is strongly associated with PHE volume after ICH. Although the exact mechanism remains unclear, it is plausible that JVR retrogradely transmits venous hypertension into the brain.

## Figures and Tables

**Table 1:** Baseline characteristics and risk factor profiles of patients with ICH.

	JVR negative	JVR positive	*p* value
Number of subjects, *n*	36	29	NA
Age in years, mean (SD)	52.9 (12.2)	55.1 (9.3)	0.422
Male gender, *n* (%)	27 (75.0)	24 (82.8)	0.449
History of smoking, *n* (%)	14 (38.9)	13 (44.8)	0.629
History of ICH, *n* (%)	0 (0.0)	4 (13.8)	0.075
History of IS, *n* (%)	4 (11.1)	3 (10.3)	NA
History of CAD, *n* (%)	2 (5.6)	2 (6.9)	NA
History of hypertension, *n* (%)	29 (80.6)	26 (89.7)	0.506
Irregular shape of hematoma, *n* (%)	5 (13.9)	9 (36%)	0.095
Admission GCS, median (IQR)	15 (13–15)	14 (12–15)	0.264
Admission mRS, median (IQR)	4 (2–4)	4 (2–5)	0.308
Admission NIHSS, mean (SD)	7.8 (5.1)	8.4 (5.4)	0.614
LDL (mmol/L), mean (SD)	3.1 (0.9)	2.8 (0.5)	0.203
GLU (mmol/L), mean (SD)	5.3 (1.4)	5.0 (1.3)	0.509
INR, mean (SD)	1.00 (0.06)	1.01 (0.04)	0.214
First SBP (mmHg), mean (SD)^†^	171 (29)	162 (24)	0.184
First DBP (mmHg), mean (SD)^†^	99 (16)	100 (15)	0.771
Baseline hematoma volume (ml), mean (SD)	19.52 (12.49)	20.29 (10.85)	0.794
sIVH, *n* (%)	6 (16.7)	5 (17.2)	1.000
Hematoma location in basal ganglia or thalamus, *n* (%)	31 (86.1)	26 (90.0)	0.958
Intraluminal structural abnormality, *n* (%)	2 (5.6)	5 (17.2)	0.268
Malformed valve	2 (5.6)	5 (17.2)	—
Flap	0	0	
Web	0	0	
Membrane	0	0	
Septum	0	0	
Extraluminal structural abnormality, *n* (%)	0	0	—

NA, not applicable; ICH, intracerebral hemorrhage; IS, ischemic stroke; CAD, coronary artery disease; GCS, Glasgow Coma Scale; mRS, modified Rankin Scale; NIHSS, National Institutes of Health Stroke Scale; LDL, low density lipoprotein; GLU, blood glucose; INR, international normalized ratio; SBP, systolic blood pressure; DBP, diastolic blood pressure; sIVH, secondary intraventricular hemorrhage; IQR, interquartile range; SD, standard deviation; ^†^first blood pressure measured after symptom observation.

**Table 2:** PHE volumes in JVR-negative and JVR-positive groups.

	JVR negative	JVR positive	*p* value
Within 72 hours			
Absolute PHE volume (ml), mean (SD)	14.69 (12.80)	23.15 (14.43)	0.015
Relative PHE volume, mean (SD)	0.74 (0.41)	1.28 (0.65)	<0.001
At 12 ± 3 days			
Absolute PHE volume (ml), mean (SD)	30.67 (23.63)	55.56 (30.34)	<0.001
Relative PHE volume, mean (SD)	2.93 (2.23)	6.83 (4.32)	<0.001

PHE, perihematomal edema; SD, standard deviation.

**Table 3:** Numbers of subjects classified based on average PHE volume.

	JVR negative	JVR positive	*p *value
Baseline absolute PHE^†^			0.041
Large-volume group, *n* (%)	12 (41.4)	1 (58.6)	
Small-volume group, *n* (%)	24 (66.7)	12 (33.3)	
Baseline relative PHE^‡^			<0.001
Large-volume group, *n* (%)	6 (24.0)	19 (76.0)	
Small-volume group, *n* (%)	30 (75.0)	10 (25.0)	
12 ± 3 days' absolute PHE^§^			0.001
Large-volume group, *n* (%)	8 (30.8)	18 (69.2)	
Small-volume group, *n* (%)	28 (71.8)	11 (28.2)	
12 ± 3 days' relative PHE^//^			0.001
Large-volume group, *n* (%)	7 (29.2)	17 (70.8)	
Small-volume group, *n* (%)	29 (70.7)	12 (29.3)	

PHE, perihematomal edema; ^†^patients classified according to average absolute PHE volume (18.47 ml) within 72 h of onset; ^‡^patients classified according to average relative PHE volume (0.98) within 72 h of onset; ^§^patients classified according to average absolute PHE volume (41.77 ml) at 12 ± 3 days after onset;  ^//^patients classified according to average relative PHE volume (4.67) at 12 ± 3 days after onset.

**Table 4:** Odd ratios in multivariate logistic regression analysis.

	OR value (95% CI)	*p *value
Based on 72 h absolute PHE^†^		
JVR	5.46 (1.04–28.63)	0.044
Baseline hematoma volume	1.14 (1.03–1.26)	0.009
Based on 72 h relative PHE^‡^		
JVR	14.85 (3.28–67.17)	<0.001
Based on absolute PHE at 12 ± 3 days^§^		
JVR	15.32 (2.52–92.99)	0.003
Baseline hematoma volume	1.14 (1.04–1.24)	0.006
Based on relative PHE at 12 ± 3 days^//^		
JVR	5.87 (1.94–17.77)	0.002

PHE, perihematomal edema; CI, confidence interval; OR, odds ratio; ^†^patients classified according to average absolute PHE volume within 72 h of onset; ^‡^patients classified according to average relative PHE volume within 72 h of onset; ^§^patients classified according to average absolute PHE volume at 12 ± 3 days after onset; ^//^patients classified according to average relative PHE volume at 12 ± 3 days after onset.
